# CA19-9 for detecting recurrence of pancreatic cancer

**DOI:** 10.1038/s41598-020-57930-x

**Published:** 2020-01-28

**Authors:** Azadeh Azizian, Felix Rühlmann, Tanja Krause, Markus Bernhardt, Peter Jo, Alexander König, Mathias Kleiß, Andreas Leha, Michael Ghadimi, Jochen Gaedcke

**Affiliations:** 1 Department of General, Visceral and Pediatric Surgery, University Medical Center Goettingen, Goettingen, Germany; 2 Department of Gastroenterology and Gastrointestinal Oncology, University Medical Center Goettingen, Goettingen, Germany; 3 Department of Interdisciplinary Oncology and Pneumology, DRK-Kliniken Nordhessen, Kassel, Germany; 4 Department of Medical Statistics, University Medical Center Göttingen, Göttingen, Germany

**Keywords:** Diagnostic markers, Outcomes research, Surgical oncology

## Abstract

CA19-9 values are regularly measured in patients with pancreatic cancer. Certainly, its potential as a biomarker has been compromised by false negative results in CA19-9 negative patients and false positive results in benign pancreatico-biliary diseases. For detection of PDAC recurrence, however, CA19-9 might play an important role. The aim of this study is to analyze the accuracy of CA19-9 for detecting recurrence of pancreatic cancer. All included patients were treated either at the University Medical Center Goettingen, or at the Department of Interdisciplinary Oncology and Pneumonology, DRK-Kliniken Nordhessen, Kassel. We analyzed data of 93 patients with pancreatic cancer in the training set and 41 in the validation set, both retrospectively. Pre- and postoperative CA19-9 values and results of imaging techniques were compared. We performed ROC-analysis. The association between longitudinally measured CA19-9 values and relapse was studied with a joint model between a random effects model for the longitudinal CA19-9 measurements and a Cox proportional hazards models for the survival data. In the test set (n = 93 patients) the median follow-up time was 644 days (22 months). Overall, 71 patients (76.3%) developed recurrence during follow-up. Patients with CA19-9 values of <10kU/l were considered as CA19-9 negative patients (n = 11) and excluded from further analysis. Among the rest, approximately 60% of the patients showed significantly elevated CA19-9 prior to detection of recurrence by imaging techniques. Recurrence was shown by 2.45 times elevated CA19-9 values with 90% positive predictive value. In the validation set, 2.45 times elevated CA19-9 values showed recurrence with 90% sensitivity and 83,33% specificity, with an area under the curve of 95%. Based on measured CA19-9 values during follow-up care, the joint model estimates in recurrence-free patients the probability of recurrence-free survival. CA19-9 elevation is an early and reliable sign for PDAC recurrence. On the strength of a very high accuracy in CA19-9 positive patients, it should be considered to use CA19-9 for therapy decision even without a correlate of imaging technics. Using the joint model, follow-up care of PDAC patients after curative therapy can be stratified.

## Introduction

Carbohydrate 19-9 antigen (CA19-9), discovered in 1982^1^, is regularly expressed on cells of the pancreatico-biliary system. It was initially detected by the monoclonal antibody 19-9^2^. In healthy individuals, it shows a low concentration in the serum (<37kU/l).

In cancer patients, serum concentration of CA19-9 is often elevated^3^. However, expression of CA19-9 is dependent on the Lewis blood group (Le): While individuals of the Le(a + b−) and Le(a−b+) blood group are capable to express CA19-9, individuals of the Le(a−b−) blood group lack the fucosyltransferase that catalyzes the synthesis of the sugar sequence^4^. About 5–7% of the population belong to the Le(a−b−) blood group and are unable to express CA19-9.

Since its discovery, CA19-9 has been analyzed in many cancer entities e.g. colorectal cancer, gastric cancer, ovarian cancer and bile duct cancer. However, its highest sensitivity and specificity is achieved in pancreatic cancer patients^5^, which makes it exceptionally valuable, given the fact that pancreatic cancer represents the seventh leading cause of cancer mortality though being only the twelfth most common malignancy worldwide^6^. Based on the expected demographic shift, pancreatic cancer (PC) is even to become the second leading cause of cancer-related death by 2030^7^. Therefore, reliable biomarkers for pancreatic cancer are highly needed and CA19-9 in this matter has been the purpose of many studies. O’Brien *et al*. showed in a retrospective analysis that serum CA19-9 is significantly upregulated up to 2 years prior to diagnosis of PC, with a specificity of 95% and a sensitivity of 53%^8^. Pre- and postoperative CA19-9 levels might even predict prognosis^9,10^. Furthermore CA19-9 levels correlate with tumor size, tumor stage and tumor burden^11^.

However, CA19-9 as a biomarker has its known limitations: Routine usage of CA19-9 as a screening tool for PC among general public is ineffective and results in a low positive predictive value due to the relatively low incidence of PC in the general population^12^. This was also shown in two large population-scale studies^13,14^). Furthermore, false positive results are observed in benign pancreatico-biliary diseases like cholangitis, pancreatitis or obstructive jaundice^15,16^. Also hepatic and pancreatic cysts might interfere with CA19-9 levels^17,18^.

Despite its limitations, CA19-9 has recently, almost three decades after its discovery, gained new interest in pancreatic cancer. Unlike earlier studies, some recent investigations suggest a more differentiated exposure to CA19-9: Luo *et al*. suggest optimizing the usage of CA19-9 by a prior Lewis and Secretor genotyping^19^. Others use the dynamic changes of CA19-9 values to monitor chemotherapy response in locally advanced or metastatic status^20,21^ or during neoadjuvant therapy^22^.

In the adjuvant setting after curative intended surgery elevation of CA19-9 during surveillance is suggestive of pancreatic cancer recurrence. However, its accuracy is still debatable since there are benign causes for postoperative CA19-9 elevation such as biliary obstruction or cholangitis that must be considered^12^. Therefore, it is recommended to diagnose recurrence of PC using imaging techniques like CT scan, endoscopy, or MRI^23^. However, these techniques have differences in accuracy, side effects, economic feasibility, reproducibility and objectivity. Furthermore, due to performed surgery the initial anatomy has changed and inflammatory residues along the superior mesenteric artery are hard to distinguish from neoplastic tissue, and therefore diagnosis of local recurrence is often delayed.

Due to the high rate of pancreatic cancer recurrence of 66-92% in the first two years after curative intended surgery and adjuvant chemotherapy^24^, biomarkers to detect the recurrence early and sufficient are still needed in the clinical practice.

For this reason, we aimed to overcome the drawbacks of static CA19-9 measurements. First we integrated postoperative values in comparison to the dynamic development of consecutive measurements to assess the presence of recurrence. Second, we used these values and their longitudinal assessment by integrating these data into a joint model to assess the likelihood of recurrence. These data are compared to the general practice of imaging and its superiority is shown.

## Methods

### Set of patients

Overall n = 134 patients were retrospectively included in this analysis. The local ethics committee approved the present study and waived the need for informed consent, due to the retrospective character of the study.

In a first set (test set) 93 patients were included with a histologically confirmed pancreatic ductal adenocarcimona (PDAC) treated at the Department of General, Visceral and Pediatric Surgery at University Medical Center Goettingen (Germany) between June 2008 and February 2015.

In a second set (independent validation set) 41 patients were included with a histologically confirmed PDAC treated either at the Department of General, Visceral and Pediatric Surgery at the University Medical Center Goettingen, Germany, or at the Department of Interdisciplinary Oncology and Pneumology, DRK-Kliniken Nordhessen, Kassel (Germany) between June 2009 and January 2017.

All patients included in this analysis received curative intended surgical resection of the tumor predominantly followed by adjuvant chemotherapy. According to the current guidelines, the adjuvant chemotherapy implied a Gemcitabine and/or 5-FU-based chemotherapy.

Ten patients received preoperative treatment with FOLFIRINOX (three cases in the training set and seven in the validation set), five of those patients were treated in line with the CONKO-007 study (EudraCT: 2009–014476-21, NCT01827553).

CA19-9 values and results of imaging techniques (mostly computed tomography scans) were compared.

We excluded in both cohorts all patients with neuroendocrine tumors, R2-resection and palliative intended surgery.

### Statistical analysis

Patients with persistent CA19-9 values < 10 kU/l were considered as CA19-9 negative patients and were excluded from further recurrence prediction analyses. For all the other patients, two analyses were performed.

First, the relative change of the postoperative values was calculated by dividing the maximum postoperative value (before image recognition for patients with relapse respectively) by the first available postoperative value. These relative changes were analyzed with respect to relapse by a receiver operating characteristic (ROC) curve. Different cutoff points as well as sensitivity, specificity and predictive values were reported. The significance level was set to α = 5% for all statistical tests. Therefore, Youden’s index was defined for all points of the ROC curve, and the maximum value of the index was used as a criterion for selecting the optimum cut-off point. All analyses were performed with the statistic software R (version 3.4.0, www.r-project.org) using the R-package ‘pROC’ for the receiver operating characteristic (ROC) analyses.

Second, a Joint Model was applied to study longitudinal CA19-9 values and relapse. Therefore, the repeated log2-scaled measurements of postoperative CA19-9 values were modeled using a random intercept and slope model. A Cox proportional hazards model was fit to the survival data. Both models were combined using a joint model which allows to study the association between the endogenous covariate CA19-9 and the risk of an event. Patient specific risk predictions have been derived from that model taking all longitudinal measurements of CA19-9 into account. Recurrence free survival (RFS; survival interval after curative intended therapy and till detection of recurrence of pancreatic cancer, in days) was estimated using Kaplan-Meier.

Both analyses were first established in the training set and consequently validated in the second patient set.

### Ethics approval and consent to participate

The presented retrospective study is approved by the local ethics comity of University Medical Center Goettingen. The ethics committee waived the need for informed consent.

## Results

### Demographic data

In the test set 93 patients were included. Of these 54 (58.1%) were male and 39 (41.9%) female. The age ranged from 28 years to 84 years with a median age of 65 years.

The validation set accounts for 41 patients. Of those 25 patients (61.0%) were male and 16 patients (39.0%) were female. The age raged from 47 to 85 years with a median age of 67 years.

Clinical data of all patients including age, sex, TNM classification (according to the 7^th^ edition of the Union International Contre le Cancer staging system (UICC)), localization of the tumor, performed surgery and application of preoperative chemotherapy is listed in Table 1.

**Table 1:** Basis clinical data. *In some patients (38 in test set and 26 in validation set) Pn state remains unknown.

		Test set (n = 93)	Validation set (n = 41)
Age (years)	Median [min -max]	65 [28–84]	67 [47–85]
Sex	female	40 (43%)	16 (39%)
	male	53 (57%)	25 (61%)
T-Status	T0/T1	3 (3%)	3 (7%)
	T2	10 (11%)	6 (14%)
	T3	71 (76%)	31 (76%)
	T4	9 (10%)	0
Nodal State	negative	19 (20%)	20 (49%)
	positive	74 (80%)	21 (51%)
Metastasis	M0	92 (99%)	40 (98%)
	M1	1 (1%)	1 (2%)
Pn-State*	Pn 0	6 (6%)	2 (5%)
	Pn 1	49 (53%)	13 (32%)
Grading*	G1	1 (1%)	1 (2%)
	G2	68 (73%)	21 (51%)
	G3	24 (26%)	8 (20%)
Resection State	R0	56 (61%)	34 (83%)
	R1	37 (39%)	7 (17%)
Tumor localization	Caput	70 (75%)	34 (83%)
	Papilla vaterii	14 (15%)	0 (0%)
	Corpus	4 (4%)	4 (10%)
	Tail	5 (5%)	3 (7%)
Surgery	Pylorus preserving partial pancreatectomy	69 (74%)	31 (76%)
	Classical partial pancreatoduodenectomy	14 (15%)	1 (2%)
	Total pancreatectomy	6 (7%)	5 (12%)
	Distal pancreatectomy	4 (4%)	4 (10%)
Preoperative chemotherapy		3 (3%)	7 (17%)

### Follow-up

In the test set (n = 93 patients) the median follow-up time was 644 days (standard deviation 626.7 days, min 69 days, max 4000 days). Seventy-one patients (76.3%) developed recurrence of PADC during follow-up (median follow up time of 606 days), while twenty-two patients (22,7%) remained recurrence-free (median follow-up time of 913 days).

**Figure 1:**
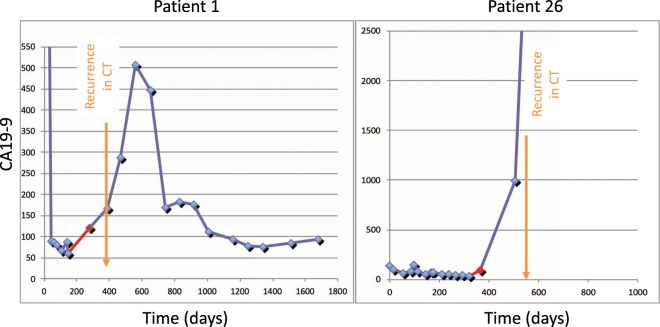
Two examples for patients with recurrence of PC during follow-up with a CA19-9 elevation prior to detection in imaging techniques (CT, computed tomography).

Eleven patients among the test set (11.8%) were considered as CA19-9 negative patients since their CA19-9 values were throughout <10kU/l – even at first diagnosis of the disease. Of the remaining 82 patients with changing CA19-9 levels, 62 patients (75.6%) developed recurrence of PDAC. Of those thirty-seven patients (59.7%) showed a significant elevation of CA19-9 prior to detection of recurrence by imaging techniques (two examples shown in Fig. 1). In 19 cases an imaging was even performed simultaneously without detecting the tumor recurrence. Here, the median time interval between significant CA19-9 elevation and evidence of cancer recurrence in imaging was 96 days (min 20 days; max 309 days).

In 19 patients (30.6%), an elevation of CA19-9 was simultaneously observed with detection of recurrence via CT or MRI. For six of 62 patients (9.7%) CA19-9 increased after detection of relapse in imaging techniques or showed no elevation at all.

Twenty-two patients of the test set showed no recurrence during surveillance (Fig. 1). Among these 14 patients (63.4%) showed elevated CA19-9 at first diagnosis, three patient (13.6%) showed normal CA19-9 values at first diagnosis (before treatment). In five patients (23%) the pretreatment CA19-9 value remains unknown.

In the validation (n = 41) the median follow-up was 628 days (standard variation 817.8 days, min value 83 days, max value 3647 days). Twenty-four patients (58.5%) developed recurrence of the disease (median follow-up time of 604 days) while seventeen patients (41.5%) remained recurrence-free during our follow-up (median follow-up time of 776 days). Thirteen patients among the validation set were considered as CA19-9 negative patients since their CA19-9 values were throughout <10kU/l.

Of the remaining thirty-one patients with changing CA19-9 levels, eighteen patients (58%) showed a relapse of PDAC during follow-up. Of those 11 patients (61.1%) showed a significant elevation of CA19-9 prior to diagnosis of recurrence via imaging techniques. For all of them imaging was even performed simultaneously without detecting the tumor recurrence. Here, the median time interval between significant CA19-9 elevation and evidence of cancer recurrence in imaging was 96.5 days (min value 45 days; max value 269 days).

A synchronous elevation of CA19-9 with the detection of recurrence in CT or MRI was observed in 4 patients (22.2%).

**Figure 2:**
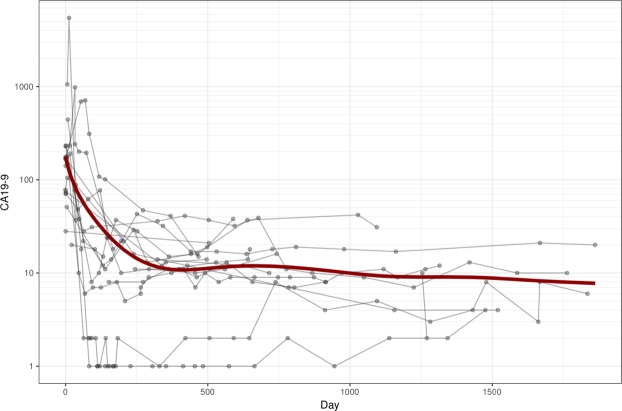
CA19-9 in recurrence-free patients during follow-up. After surgery, the initially elevated CA19-9 value drops under 50 kU/l. One patient received neoadjuvant chemotherapy. Here, chemoradiotherapy started on day 15 till day 225, surgery was performed on day 262.

In both test and validation set, none of the patients without recurrence showed an elevated CA19-9 > 50 kU/l (Fig. 2).

**Figure 3:**
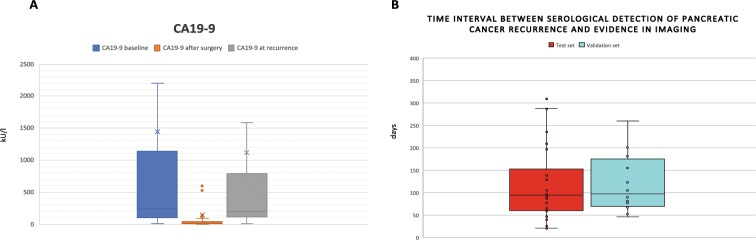
**(A)** CA19-9 values over all patients at baseline (before any therapy), after surgery (but before adjuvant chemotherapy), and at time point of recurrence are illustrated. CA19-9 negative patients are excluded. (**B)** Time interval between serological detection and evidence of recurrence in imaging in test set (red) and validation set (light blue).

The median CA19-9 values at baseline (before any treatment), after surgery and before adjuvant chemotherapy, and at time point of (serological) recurrence are shown in Fig. 3A. Overall, in 98 of 134 patients, CA19-9 level dropped to normal values (<37kU/l) after curative intended surgery. The time interval (in days) between significant CA19-9 elevation and detection of the tumor in imaging for the test set and validation set is illustrated in Fig. 3B.

Overall survival and Recurrence-free survival of both cohorts are shown in supplementary figure 1 A and B.

### CA19-9 elevation predicts recurrence of PC

**Figure 4:**
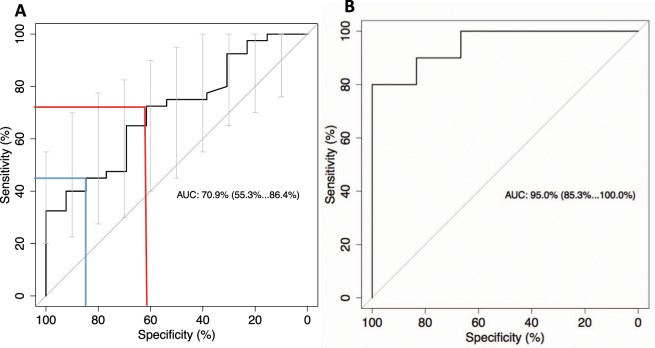
**(A)** Initial set. Analyses of relative changes of CA19-9 values during follow-up with respect to relapse by a receiver operating characteristic (ROC) curve. Two different cutoff points with differences in sensitivity, specificity and predictive values are marked. 1.35xtimes elevation of CA19-9 shows recurrence with 72% sensitivity, 62% specificity, 85% positive predictive value and 42% negative predictive value (blue line). A 2.45 x times elevation of CA19-9 shows recurrence with 45% sensitivity, 85% specificity, 90% positive predictive value and 33% negative predictive value (red line). **(B)** Independent validation set. Analyses of relative changes of CA19-9 values during follow-up with respect to relapse by a receiver operating characteristic (ROC) curve. 1.35 x times elevation of CA19-9 shows recurrence with 100% sensitivity, 67% specificity, 83% positive predictive value and 100% negative predictive value. 2.45 x times elevation of CA19-9 shows recurrence with 90% sensitivity, 83,33% specificity.

All data of CA 19-9 positive patients were analyzed concerning the development of CA19-9 and detection of the disease in CT or MRI by performing ROC analysis. At 1.35 the maximum value of Youden’s index (Y = sensitivity + specificity −1) for the ROC curve was achieved. We could demonstrate that a 1.35 times elevated CA19-9 shows recurrence with 72% sensitivity, 62% specificity, 85% positive predictive value and 42% negative predictive value. Since clinical application of biomarkers for detection of cancer relapse demands high specificity (even at the expense of a lower sensitivity), we chose a second cut-off which achieved a specificity of >80%: 2.45 times elevated CA19-9 values show recurrence with 45% sensitivity, 85% specificity, 90% positive predictive value and 33% negative predictive value (see Fig. 4).

These to cut-offs were tested in a validation set. Here 1.35 times elevated CA19-9 values predict recurrence with 100% sensitivity, 67% specificity, 83% positive predictive value and 100% negative predictive value. 2.45 times elevated CA19-9 values shows recurrence of PC with 90% sensitivity and 83,33% specificity, which an Area under the curve of 95%(see Fig. 4).

#### Progression-free survival

**Figure 5:**
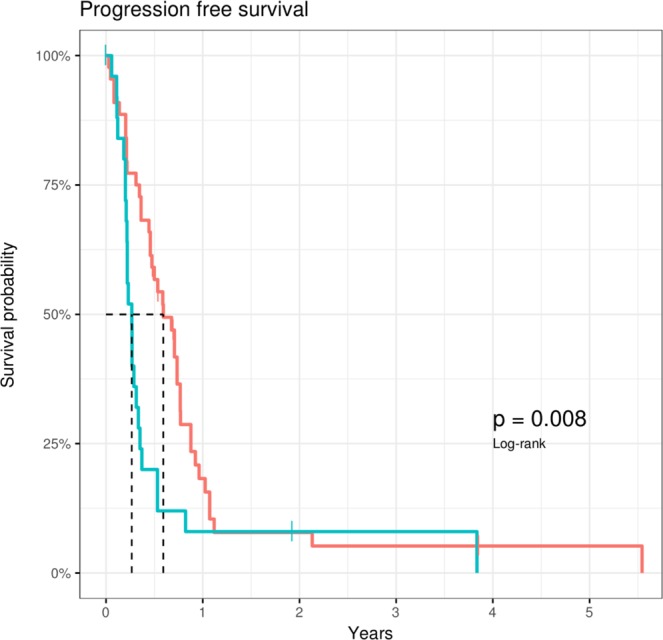
Progression-free survival (PFS) during first line palliative chemotherapy in patients with an elevation of CA19-9 prior to detection of cancer recurrence (red line) versus PFS of patients with no prior CA19-9 elevation. The p-value is 0.008.

Progression-free survival (PFS) during first line chemotherapy was compared between patients who showed a significant CA19-9 elevation prior to imaging evidence versus PFS of patients without a prior CA19-9 elevation. Here, median PFS of the first group was 97 days (95%CI; 79–128), while median PFS of the second group was 280 days (95% CI; 167–280). Figure 5 illustrates these results in a Kaplan-Meier-Curve.

### Joint model

**Figure 6:**
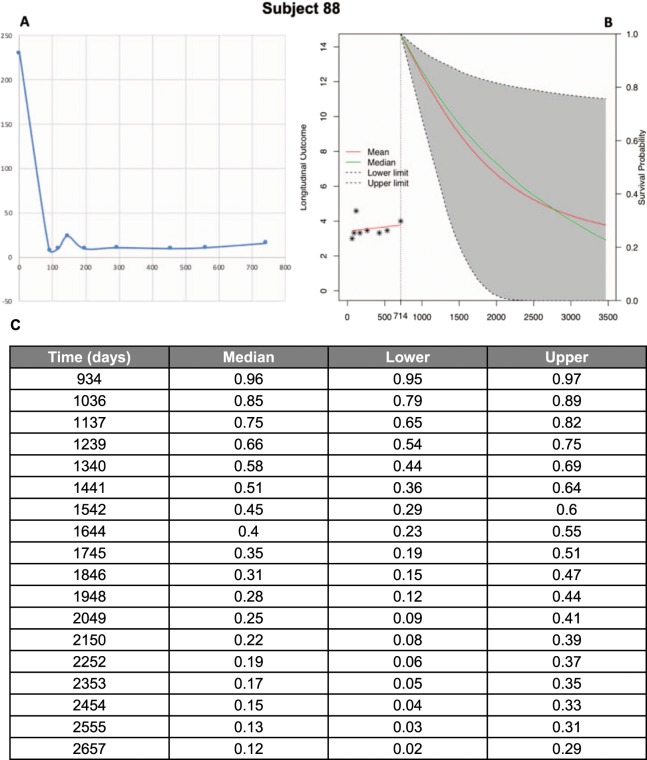
(**A)** Development of CA19-9 values of Patient 88 as an example for recurrence-free patients. **(B)** Based on measured CA19-9 values during follow-up, the joint model estimates in recurrence-free patients the probability of recurrence-free survival in the future. **(C)** The column “Time” represents the days after first diagnosis and the columns “Median”, “Lower” and “Upper” describe the probabilities of recurrence-free survival for this patients on that day. With every additional CA19-9 value measured the probabilities adjust.

According to the joint model a unit increase in log2(CA19-9) is associated with a 1.5 -fold increase in risk (95% CI: 1.38–1.65, p < 0.0001). Based on measured CA19-9 values during follow-up, the joint model estimates the probability of recurrence-free survival for recurrence-free patients. With every additional CA19-9 value measured the probabilities adjust. Figure 6 shows an example of the estimated probabilities.

**Figure 7:**
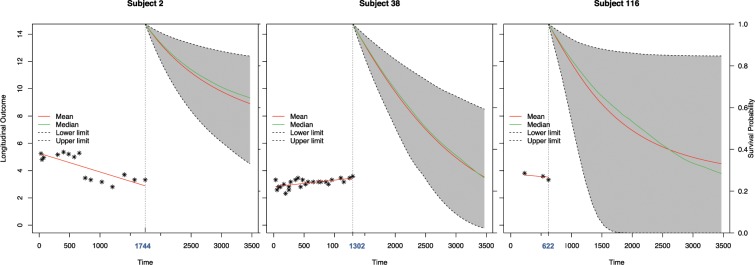
Exemplary demonstration of the estimated recurrence free survival in case of three different subjects applying the joint model. Subject 2 has a comparatively good recurrence free survival (RFS) probability; subject 38 has a poorer prognosis. Subject 116 as an example for a patient with only few CA19-9 measurements shows a wide range between lower and higher probability of RFS.

The accuracy of the joint model was validated in a second set (Fig. 7): For each patient in the validation cohort at each measurement of CA19-9 the following two months interval was analyzed.

**Figure 8:**
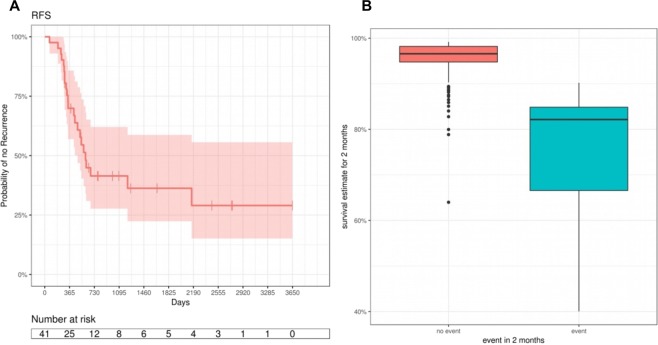
(**A)** Kaplan-Meier Curve of the validation set using the Joint Model. Recurrence free survival time was estimated using Kaplan-Meier. (**B)** Estimated recurrence free survival probabilities (RFS) in the validation data.

The validation data contains 14 events within two months of a CA19-9 measurement. Two months survival estimates were predictive for events within that time (AUC = 0.98). For predicting an event for all cases with an estimated 2 months survival probability smaller than 90.3%, the sensitivity was 1, i.e. 100% of the events were correctly predicted, and the specificity was 92% of the non-events were correctly predicted (see Fig. 8).

## Discussion

For several years now CA19-9 measurements are used to monitor the effectiveness of chemotherapy in advanced pancreatic cancer^20,21^. However, in the postoperative follow-up after resection of pancreatic cancer, the clinical relevance of CA19-9 is regarded as very low. Currently, diagnosis of recurrence is based on imaging techniques. While elevated CA19-9 values raise concern of a cancer relapse, no treatment takes place until the appearance of a correlate in the CT/MRI scan. Nevertheless, it is evident that imaging techniques have a clear limitation in detecting pancreatic cancer recurrence, if the mass is small or scatter. Furthermore, the diagnosis of peritoneal carcinomatosis or local relapse at the superior mesenteric artery is difficult and very often indirect signs of progression of the disease help to confirm diagnosis. Inevitably, this leads to a delay in starting relevant palliative chemotherapy. However, from clinical experience, CA19-9 appears to be a relevant marker and based on the idea to use the dynamic of CA19-9 instead of rigid cut-off levels we aimed to evaluate the relevance of CA19-9 again.

We assumed that the postoperative CA19-9 level varies from patient to patient and might depend on the resection status as well as on the tumor biology. This value, however, best reflects the CA19-9 baseline. We revealed that an increase of 1.35 fold already identifies a decent number of patients who did not have a visualized tumor relapse. Increasing the threshold to 2.45 fold improved the specificity to 83,3%, however, the sensitivity decreased slightly. From the clinical point of view these data allow the identification of a pancreatic cancer relapse prior to a standard imaging technique. Up to 6 months in advance CA19-9 was elevated while CT or MRI did not help to identify tumor recurrence (see Fig. 3B). Accordingly, the relapse is evident at the time point of increased CA19-9, but just not visible. As a first evidence that palliative treatment based on CA19-9 levels may be useful, Li *et al*. analyzed 80 pancreatic cancer patients. 32.5% or the patients received chemotherapy before tumor was visible. Li *et al*. suggest starting salvage treatment after pancreatic cancer resection, if CA19-9 values elevate during surveillance^25^. They split up the patients according to the imaging results and were able to show an improved DFS and OS for those patients with an initiation of therapy before imaging showed the relapse. In our own data set, we analyzed the PFS during first line chemotherapy after detection of recurrence in imaging. We could show that those patients with a significant CA19-9 elevation prior to imaging had a worse median PFS compared to those who showed a simultaneous CA19-9 elevation and detection of PDAC relapse in imaging (98 days vs. 182 days in training set; 102 days versus 228 days). We argue, that this might be the result of delayed palliative chemotherapy.

With respect to the improved results in the validation group and the higher accuracy of CA19-9 elevation to detect PC recurrence with an AUC of 90% (AUC of the initial set: 70.9%) it becomes evident that the number of CA19-9 measurements were higher in the validation group. This may point out that regular and frequent CA19-9 measurements help to improve the accuracy of recurrence prediction. Nevertheless we should consider that partially the beneficial effect of CA19-9 measurements might be determined be the surveillance protocol. In some cases of this study, the earliness CA19-9 elevation was possibly due to the lack of imaging. However, in 30 of 48 cases with prior significant CA19-9 elevation, a simultaneous imaging was even performed, but did not detect the cancer recurrence.

Overall, it is evident that the use of a stringent cut-off level for all pancreatic cancer patients leads to results that certainly justify not using CA19-9 as a relevant maker for disease control. However, adjusting these data with personal, postoperative values allows a highly sensitive and specific prediction of recurrence.

Although the stringent measurement of CA19-9 helps to identify relapse in pancreatic cancer and this may translate into better survival it should be kept in mind that time intervals of follow-up assessment are increasing after surgery. Even more, there is no standardized strategy of frequency of imaging analyses. To further explore this problem we used the different CA19-9 in a joint model. This model is a statistical tool to effectively estimate treatment effects on the time to event. It well serves for longitudinal markers and is already used in clinical trials and observational studies. It has several advantages compared to conventional models (e.g., a Cox model) as shown by Ibrahim *et al*.^26^. Applied to our data set we were able to show that a relapse probability can be estimated by a high accuracy. As surveillance of patients without any evidence of recurrence are followed in expanding time intervals, these data now allow to individualize the follow-up. In combination with the knowledge from the above discussed increased relevance of CA 19-9 levels it is possible to either start early treatment of assumed recurrence or at least to determine the time point of a CT or MRI, instead of waiting for routine analyses or a symptomatic disease.

Overall, we are not the first to emphasize the relevance of CA 19-9 elevation. Motoi *et al*. recently showed that CA19-9 elevation is a strong predictor of hepatic recurrence^27^. In our analysis there was no difference between hepatic, pulmonary or local recurrence concerning CA19-9 accuracy.

### Limitation of the study

The present work is a retrospective study of two independent cohorts and underlies several limitations. In some cases the baseline CA19-9 value remains unknown, the intervals between CA19-9 measurements as well as between two imaging applications differs widely since no standard surveillance protocol was applied. Also the exact courses of adjuvant chemotherapy, its starting point and last day of chemotherapy remains partially unclear.

## Conclusion

We here present a dynamic approach to use CA19-9 values for detecting pancreatic cancer recurrence reliably. On the strength of a very high accuracy in CA19-9 positive patients, it should be considered to use CA19-9 for therapy decision even without a correlate of imaging technics. Plus, it can be used for stratification of surveillance avoiding unnecessary CTs and MRIs for low-risk patients on the one hand and applying more frequent checks and test for high-risk patients on the other hand.

## Supplementary information

Supplementary information.

## Data Availability

The datasets used and/or analyzed during the current study are available from the corresponding author on reasonable request.
