# Supplementary material for: CA19-9 for detecting recurrence of pancreatic cancer
Source: Sci Rep. 2020 Jan 28;10:1332. doi: 10.1038/s41598-020-57930-x (PMC6987233; doi:10.1038/s41598-020-57930-x)

CA19-9 for detecting recurrence of pancreatic cancer

Azadeh Azizian[1], Felix Rühlmann[1], Tanja Krause[1], Markus Bernhardt[1], Peter Jo[1], Alexander König[2], Mathias Kleiß[3], Andreas Leha[4], Michael Ghadimi[1], and Jochen Gaedcke[1*].

[1] Department of General, Visceral and Pediatric Surgery, University Medical Center Goettingen, Goettingen, Germany.

[2] Department of Gastroenterology and Gastrointestinal Oncology, University Medical Center Goettingen, Goettingen, Germany.

[3] Department of Interdisciplinary Oncology and Pneumology, DRK-Kliniken Nordhessen, Kassel, Germany

[4] Department of Medical Statistics, University Medical Center Göttingen, Göttingen, Germany.

<u>Supplementary tables' legends</u>

Supplementary table S1: Youden index (Youden), sensitivity, specificity, positive predictive value (PPV) and negative predictive value (NPV) for all points of the ROC curve in figure 3A (initial set).

Supplementary table S2: Youden index (Youden), sensitivity, specificity, positive predictive value (PPV) and negative predictive value (NPV) for all points of the ROC curve in figure 3B (validation

Supplementary figure 1: (A) Overall survival for test set (red) and validation set (light blue) as Kaplan-Meier-Curve. **(B)** Recurrence-free survival set (red) and validation set (light blue) as Kaplan-Meier-Curve.

Supplementary table S1

Threshold	Sensitivity	Specificity	PPV	NPV	Youden
1	82.5	30.77	78.57	36.36	13.27
1.1	77.5	38.46	79.49	35.71	15.96
1.2	75	38.46	78.95	33.33	13.46
1.3	75	53.85	83.33	41.18	28.85
**1.35**	**72.5**	**61.54**	**85.29**	**42.11**	**34.04**
1.4	70	61.54	84.85	40	31.54
1.45	70	61.54	84.85	40	31.54
1.5	67.5	61.54	84.38	38.1	29.04
1.55	65	61.54	83.87	36.36	26.54
1.6	65	61.54	83.87	36.36	26.54
1.7	62.5	69.23	86.21	37.5	31.73
1.8	*60*	69.23	85.71	36	29.23
1.9	60	69.23	85.71	36	29.23
2	60	69.23	85.71	36	29.23
2.05	60	69.23	85.71	36	29.23
2.1	55	69.23	84.62	33.33	24.23
2.15	55	69.23	84.62	33.33	24.23
2.2	52.5	69.23	84	32.14	21.73
2.25	47.5	69.23	82.61	30	16.73
2.3	47.5	76.92	86.36	32.26	24.42
2.35	45	76.92	85.71	31.25	21.92

Threshold	Sensitivity	Specificity	PPV	NPV	Youden
2.4	45	84.62	90	33.33	29.62
**2.45**	**45**	**84.62**	**90**	**33.33**	**29.62**
2.5	45	84.62	90	33.33	29.62
2.6	42.5	84.62	89.47	32.35	27.12
2.7	42.5	84.62	89.47	32.35	27.12
2.8	42.5	84.62	89.47	32.35	27.12
2.9	40	84.62	88.89	31.43	24.62
3	40	84.62	88.89	31.43	24.62
3.1	37.5	92.31	93.75	32.43	29.81
3.2	37.5	92.31	93.75	32.43	29.81
3.3	35	92.31	93.33	31.58	27.31
3.4	35	92.31	93.33	31.58	27.31
3.5	32.5	92.31	92.86	30.77	24.81
3.6	32.5	92.31	92.86	30.77	24.81
3.7	32.5	92.31	92.86	30.77	24.81
3.8	32.5	92.31	92.86	30.77	24.81
3.9	32.5	92.31	92.86	30.77	24.81
4	32.5	92.31	92.86	30.77	24.81
4.05	32.5	100	100	32.5	32.5

Supplementary table S2

Threshold	Sensitivity	Specificity	PPV	NPV	Youden
1	100	16.67	66.67	100	16.67
1.1	100	16.67	66.67	100	16.67
1.2	100	33.33	71.43	100	33.33
1.3	100	50	76.92	100	50
**1.35**	100	66.67	83.33	100	66.67
1.4	100	66.67	83.33	100	66.67
1.45	100	66.67	83.33	100	66.67
1.5	100	66.67	83.33	100	66.67
1.55	90	66.67	81.82	80	56.67
1.6	90	83.33	90	83.33	73.33
1.65	90	83.33	90	83.33	73.33
1.7	90	83.33	90	83.33	73.33
1.75	90	83.33	90	83.33	73.33
1.8	90	83.33	90	83.33	73.33
1.85	90	83.33	90	83.33	73.33
1.9	90	83.33	90	83.33	73.33
1.95	90	83.33	90	83.33	73.33
2	90	83.33	90	83.33	73.33
2.1	90	83.33	90	83.33	73.33
2.2	90	83.33	90	83.33	73.33
2.3	90	83.33	90	83.33	73.33

Threshold	Sensitivity	Specificity	PPV	NPV	Youden
2.4	90	83.33	90	83.33	73.33
2.45	45	84.62	90	83.33	73.33
2.5	90	83.33	90	83.33	73.33
2.6	90	83.33	90	83.33	73.33
2.7	80	83.33	88.89	71.43	63.33
2.75	80	83.33	88.89	71.43	63.33
2.8	80	83.33	88.89	71.43	63.33
2.85	80	83.33	88.89	71.43	63.33
2.9	80	83.33	88.89	71.43	63.33
2.95	80	83.33	88.89	71.43	63.33
3	80	83.33	88.89	71.43	63.33
3.05	80	100	100	75	80

Supplementary figure 1

A

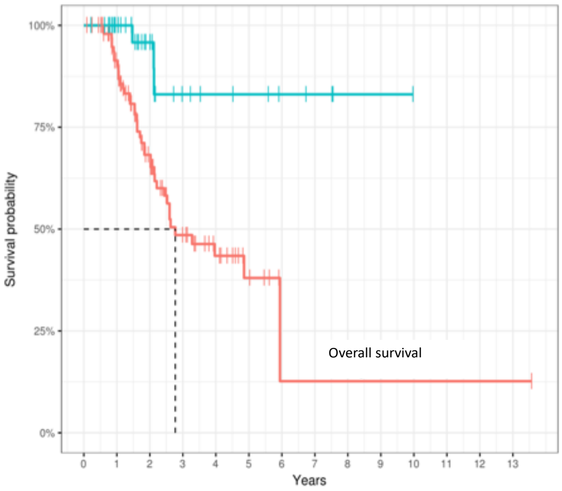

B

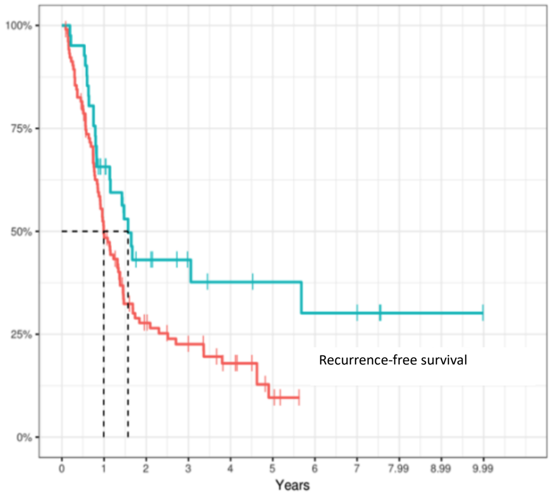

Supplement: Supplementary file 1 — Supplementary information. [file 41598_2020_57930_MOESM1_ESM.pdf]
